# Regional Differences and Similarities in Diabetes Care in Japan: Insights from the J-DOME Registry

**DOI:** 10.31662/jmaj.2025-0152

**Published:** 2025-09-26

**Authors:** Mitsuhiko Noda, Kohjiro Ueki, Atsushi Goto, Koichi Node, Hiromi Rakugi, Narumi Eguchi

**Affiliations:** 1 Department of Diabetes, Metabolism and Endocrinology, Ichikawa Hospital, International University of Health and Welfare, Chiba, Japan; 2 Department of Endocrinology and Diabetes, Saitama Medical University, Saitama, Japan; 3 Diabetes Research Center, Japan Institute for Health Security, Tokyo, Japan; 4 Department of Public Health, School of Medicine, Yokohama City University, Kanagawa, Japan; 5 Department of Cardiovascular Medicine, Saga University, Saga, Japan; 6 Osaka Rosai Hospital, Osaka, Japan; 7 Japan Medical Association Research Institute, Tokyo, Japan

**Keywords:** diabetes care, regional difference in Japan, diabetes specialist, diabetes non-specialist, primary care institution, patient registry research

## Abstract

**Introduction:**

Comparing diabetes care across different regions of Japan is essential for informing future healthcare policy. Additionally, since many patients with diabetes receive treatment from non-specialist physicians, it is important to determine whether differences exist between diabetes specialists and non-specialists in terms of medical care and to identify specific areas, if any, where these differences occur.

**Methods:**

To investigate this, we utilized data from J-DOME (Japan Medical Association Database of Clinical Medicine), a patient registry established as a nationwide project by the Japan Medical Association. Patients with type 2 diabetes were categorized into seven regional groups based on Japan’s prefectural divisions, and a regional comparison was conducted. Differences between specialists and non-specialists were also examined.

**Results:**

A total of 116 medical institutions encompassing 2,938 patients were included in the analysis. The nationwide mean glycated hemoglobin level was 6.96% (standard deviation [SD]: 0.46), with no statistically significant regional variations. Similarly, the nationwide mean blood pressure was 129.7/73.0 mmHg (SD: 6.1/5.7 mmHg), with no significant regional differences observed.

The average rates of regular ophthalmologic visits and urinary albumin quantification testing were 63.7% (SD: 31.3) and 40.2% (SD: 38.4), respectively. These rates were significantly higher in institutions led by diabetes specialists compared to those managed by non-specialists (regular ophthalmologic visit rate: non-specialists institutions: 53.9% [SD: 33.8]; diabetes specialist institutions: 78.5% [SD: 19.5], p < 0.001; urinary albumin quantification test rate among patients without macroproteinuria: non-specialist institutions: 33.5% [SD: 39.0]; diabetes specialist institutions: 62.5% [SD: 35.5], p < 0.001).

**Conclusions:**

This survey revealed no significant regional differences in diabetes care. However, certain aspects of diabetes management differed significantly between diabetes specialists and non-specialist physicians.

## Introduction

The global population of patients with diabetes is increasing annually. The International Diabetes Federation estimates that, in 2024, the number of people with diabetes will reach 589 million ^(1)^. In Japan, the Ministry of Health, Labour and Welfare reported in the National Health and Nutrition Survey 2019 that 19.7% of males and 10.8% of females were suspected to have diabetes, representing approximately one-seventh of the Japanese population ^(2)^.

Complications of diabetes mellitus not only worsen prognosis but also reduce quality of life, with the high cost of treatment being a significant social issue. Effective diabetes management, including long-term control of blood glucose, body weight, blood pressure, and lipids, is expected to prevent the onset and progression of microvascular (neuropathy, retinopathy, nephropathy) and macrovascular complications, thereby promoting a longer and healthier life.

Given the large number of individuals with diabetes, understanding the state of diabetes care in Japan, including regional disparities, is crucial. This aligns with the objectives outlined in *Health Japan 21 (Third Phase)*, which aims to extend healthy life expectancy and reduce health disparities ^(3)^. Preventing the progression of lifestyle-related diseases, such as diabetes, and addressing regional disparities in their management are essential.

Despite the high prevalence of diabetes, only 7,150 diabetes specialists were certified by the Japan Diabetes Society as of January 6, 2025 ^(4)^, suggesting that many patients with diabetes receive care from non-specialist physicians. Therefore, investigating potential differences in clinical management between specialists and non-specialists is vital.

In this study, we utilized the Japan Medical Association Database of Clinical Medicine (J-DOME), a registry of patients with lifestyle-related diseases established through the nationwide *Primary Care Physician Clinical Database Research Project* of the Japan Medical Association ^(5)^. By conducting a regional comparison of registered patients, we aimed to assess regional variations in diabetes care and contribute to the development of strategies to improve the management of diabetes and other lifestyle-related diseases.

## Materials and Methods

### Study design and participants

This cross-sectional analysis targeted primary care institutions that registered patients in the Japan Medical Association Database of Clinical Medicine (J-DOME) during the 2022 fiscal year (registration period: April 2022-May 2023). J-DOME is a patient registry system managed by the Japan Medical Association, in which physicians at medical institutions voluntarily input their patients’ data.

J-DOME began enrolling patients with type 2 diabetes, hypertension, dyslipidemia, and chronic kidney disease in 2018, 2020, and 2022, respectively. Inclusion criteria were as follows: (1) patients diagnosed with type 2 diabetes, hypertension, dyslipidemia, or chronic kidney disease by a physician; (2) patients receiving treatment at primary care institutions (clinics or small-to-medium-sized hospitals) in Japan; and (3) no age or sex restrictions.

Participants in the J-DOME were selected by their primary care physicians and registered after obtaining verbal informed consent. For this study (2022 fiscal year cross-sectional analysis), the analysis set consisted of patients registered as having type 2 diabetes, with the medical institutions as the unit of analysis. Inclusion criteria for medical institutions were as follows: (1) institutions participating in J-DOME and (2) institutions that registered patients in 2022. No exclusion criteria were defined. To reduce variability in the number of cases per institution, a maximum of 150 cases per facility were included in the analysis, with selection based on the earliest registration dates at each institution.

### Regional classification

In this study, medical institutions were classified into seven regions based on their address:

1. Hokkaido/Tohoku: Hokkaido, Aomori, Iwate, Miyagi, Akita, Yamagata, Fukushima

2. Northern Kanto: Ibaraki, Tochigi, Gunma, Saitama

3. Southern Kanto: Chiba, Tokyo, Kanagawa

4. Chubu: Niigata, Toyama, Ishikawa, Fukui, Yamanashi, Nagano, Gifu, Shizuoka, Aichi

5. Kinki: Mie, Shiga, Kyoto, Osaka, Hyogo, Nara, Wakayama

6. Chugoku/Shikoku: Tottori, Shimane, Okayama, Hiroshima, Yamaguchi, Tokushima, Kagawa, Ehime, Kochi

7. Kyushu/Okinawa: Fukuoka, Saga, Nagasaki, Kumamoto, Oita, Miyazaki, Kagoshima, Okinawa.

### Definition of specialist institutions

This study compared *specialist institutions* and *non-specialist institutions*. A specialist institution was defined as a medical facility where a physician certified as a diabetes specialist by the Japan Diabetes Society provided medical care.

### Outcome measures for statistics

The following clinical parameters were analyzed: glycated hemoglobin (HbA1c) based on the National Glycohemoglobin Standardization Program standards, systolic blood pressure, diastolic blood pressure, low-density lipoprotein (LDL) cholesterol, high-density lipoprotein (HDL) cholesterol, triglycerides, regular ophthalmologic visit rate, and urinary albumin quantification test rate.

Clinical data were measured and recorded at each primary care institution by the attending physician and subsequently registered in J-DOME. The most recent available data were used for analysis. Data were aggregated at the institution level, with laboratory values calculated as arithmetic means for each institution. Rates of regular ophthalmologic visits and urinary albumin quantification tests were also calculated per institution. As such, the average clinical indices, including test values, for patients at each facility were calculated and used as the representative values for that facility. In other words, each facility (i.e., medical institution) was treated as a single observation unit. Subsequently, the overall and region-specific averages were calculated at the facility level. At the same time, the variability in the representative values across facilities was expressed as the standard deviation (SD) for both overall and region-specific data. Since triglyceride levels did not follow a normal distribution and instead approximated a log-normal distribution, log-transformed values were used for statistical calculations.

### Statistical analysis

The sample included all medical institutions that registered cases in J-DOME during the 2022 fiscal year.

Descriptive statistics were calculated for all institutions, by region, and by specialist/non-specialist classification. Summary statistics included the mean, SD, median, and interquartile range.

To evaluate the heterogeneity in facility-representative laboratory values and regular examination rates between regions, the *I*^2^ statistic was calculated, and Cochran’s Q test was performed.

All statistical analyses were conducted using SPSS (version 24.0, IBM Japan, Ltd.).

## Results

### Study population

A total of 125 medical institutions, comprising 3,987 patients, were included in the analysis. The median age of these patients was 72 years (range: 8-99 years), with 2,286 males and 1,701 females. The mean number of patients per institution was 31.9 (range: 1-150). Of these, 2,938 patients diagnosed with type 2 diabetes were extracted and included in the current analysis. Descriptive statistics for this analysis set (patients with type 2 diabetes) are presented in Table 1. The median age of these patients was 71 years (range: 18-99 years), with 1,567 males and 1,371 females. The mean number of patients per institution was 25.3 (range: 1-150).

**Table 1.** Descriptive Statistics of the Analysis Set Population.

Statistics Item/Category/Region	Statistics
By medical institution unit	
Number of institutions (n)	116
Number of patients registered per institution (n)	
Mean (SD)	25.3 (29.8)
Median [IQR]	15.5 [7.0-30.0]
Range (min-max)	1-150
Specialist institutions/Non-specialist institutions (n)	45/71
Region (n)	
Hokkaido and Tohoku	11
North Kanto	13
South Kanto	32
Chubu	18
Kinki	11
Chugoku and Shikoku	19
Kyushu and Okinawa	12
By diabetes patient unit	
Number of patients (n)	2,938
Gender (male/female)	1,772/1,166
Age (years)	
Mean (SD)	69.2 (11.9)
Median [IQR]	71.0 [62.0-78.0]
Range	18-99
Specialist institutions/Non-specialist institutions (n)	1,567/1,371
Region (n)	
Hokkaido and Tohoku	119
North Kanto	483
South Kanto	1138
Chubu	231
Kinki	373
Chugoku and Shikoku	375
Kyushu and Okinawa	219

IQR: inter-quartile range; SD: standard deviation.

The regional distribution of participating institutions was as follows: (1) Hokkaido/Tohoku: 11 institutions (119 patients), (2) Northern Kanto: 13 institutions (483 patients), (3) Southern Kanto: 32 institutions (1,138 patients), (4) Chubu: 18 institutions (231 patients), (5) Kinki: 11 institutions (373 patients), (6) Chugoku/Shikoku: 19 institutions (375 patients), and (7) Kyushu/Okinawa: 12 institutions (219 patients).

Of the 116 institutions with patients with type 2 diabetes, 45 (1,567 patients) were classified as specialist institutions, and 71 (1,371 patients) were non-specialist institutions. Among these patients, 22.8% of those treated at specialist institutions and 7.7% at non-specialist institutions were with diabetic retinopathy (15.7% overall, p < 0.001 between groups). Similarly, diabetic nephropathy was observed in 21.8% of patients at specialist institutions and 16.1% at non-specialist institutions (19.1% overall, p < 0.001 between groups).

### Regional differences in clinical practices

The mean clinical parameters by region are summarized in Table 2. The overall mean HbA1c level across all institutions was 6.96% (SD: 0.46). Similarly, the mean systolic blood pressure was 129.7 mmHg (SD: 6.1) and the mean diastolic blood pressure was 73.0 mmHg (SD: 5.7), with no significant regional differences (systolic blood pressure: *I*^2^ = 25.89%, p = 0.216; diastolic blood pressure: *I*^2^ = 0.00%, p = 0.682).

**Table 2.** Regional Aggregation of Mean Clinical Laboratory Values by Medical Institution Unit.

Region	Mean	SD	Median	IQR	*I^2^*	p Value
HbA1c [NGSP] (%)					0.01%	0.242
All regions	6.96	0.46	7.0	6.7-7.2		
Hokkaido and Tohoku	6.53	0.63	6.7	6.1-7.1		
North Kanto	7.10	0.42	7.1	6.8-7.2		
South Kanto	7.03	0.35	7.0	6.8-7.3		
Chubu	6.97	0.41	7.0	6.7-7.1		
Kinki	7.00	0.55	7.1	6.5-7.4		
Chugoku and Shikoku	6.92	0.50	7.0	6.6-7.3		
Kyushu and Okinawa	7.07	0.35	7.1	6.8-7.4		
Systolic blood pressure (mmHg)					25.89%	0.216
All regions	129.7	6.1	130	126-134		
Hokkaido and Tohoku	130.4	6.4	131	127-134		
North Kanto	128.1	6.0	129	122-133		
South Kanto	130.7	4.6	130	128-134		
Chubu	129.2	6.8	128	125-133		
Kinki	129.5	6.7	129	125-134		
Chugoku and Shikoku	131.7	5.4	132	128-135		
Kyushu and Okinawa	125.7	7.5	124	120-129		
Diastolic blood pressure (mmHg)					0.00%	0.682
All regions	73.0	5.7	73	69-77		
Hokkaido and Tohoku	76.8	9.8	74	72-81		
North Kanto	72.5	6.1	72	66-79		
South Kanto	72.1	4.0	73	69-74		
Chubu	73.7	4.9	73	72-77		
Kinki	72.9	6.1	72	68-79		
Chugoku and Shikoku	73.2	5.4	73	69-77		
Kyushu and Okinawa	71.7	5.5	70	67-77		
LDL cholesterol (mg/dL)					13.00%	0.302
All regions	105.6	12.5	105	97-111		
Hokkaido and Tohoku	98.5	12.7	103	91-107		
North Kanto	104.4	13.9	106	98-115		
South Kanto	106.2	11.5	105	98-114		
Chubu	102.7	9.1	104	94-108		
Kinki	109.1	15.7	108	95-123		
Chugoku and Shikoku	109.7	13.2	109	103-111		
Kyushu and Okinawa	105.4	12.4	101	97-113		
HDL cholesterol (mg/dL)					0.00%	0.477
All regions	57.6	6.1	58	54-60		
Hokkaido and Tohoku	58.1	5.6	58	53-62		
North Kanto	54.4	5.8	55	53-58		
South Kanto	57.2	4.6	57	55-59		
Chubu	59.2	5.8	59	55-63		
Kinki	58.7	6.8	59	52-63		
Chugoku and Shikoku	57.6	7.9	58	53-61		
Kyushu and Okinawa	58.0	7.3	57	54-59		
log Triglyceride (mg/dL)*					3.11%	0.682
All regions	2.11	0.10	146	126-168		
Hokkaido and Tohoku	2.10	0.16	126	111-181		
North Kanto	2.12	0.11	148	122-179		
South Kanto	2.10	0.06	146	130-163		
Chubu	2.15	0.12	149	128-198		
Kinki	2.09	0.12	149	122-159		
Chugoku and Shikoku	2.13	0.09	143	131-176		
Kyushu and Okinawa	2.11	0.07	152	122-166		

p-value: Test for heterogeneity between regions (Cochran’s Q test). *The median and IQR for triglycerides are displayed as inverse log-transformed values (original scale). IQR: interquartile range [25%-75%]; SD: standard deviation.

For lipid metabolism markers, the mean LDL cholesterol level was 105.6 mg/dL (SD: 12.5), and the mean HDL cholesterol level was 57.6 mg/dL (SD: 6.1). The mean triglyceride level, expressed as a log-transformed value, was 2.11 (SD: 0.10), which corresponds to an actual measurement of 129.5 mg/dL. No significant regional differences were observed for these markers (LDL cholesterol: *I*^2^ = 13.00%, p = 0.302; HDL cholesterol: *I*^2^ = 0.00%, p = 0.477; triglycerides: *I*^2^ = 3.11%, p = 0.682).

**Figure 1.**
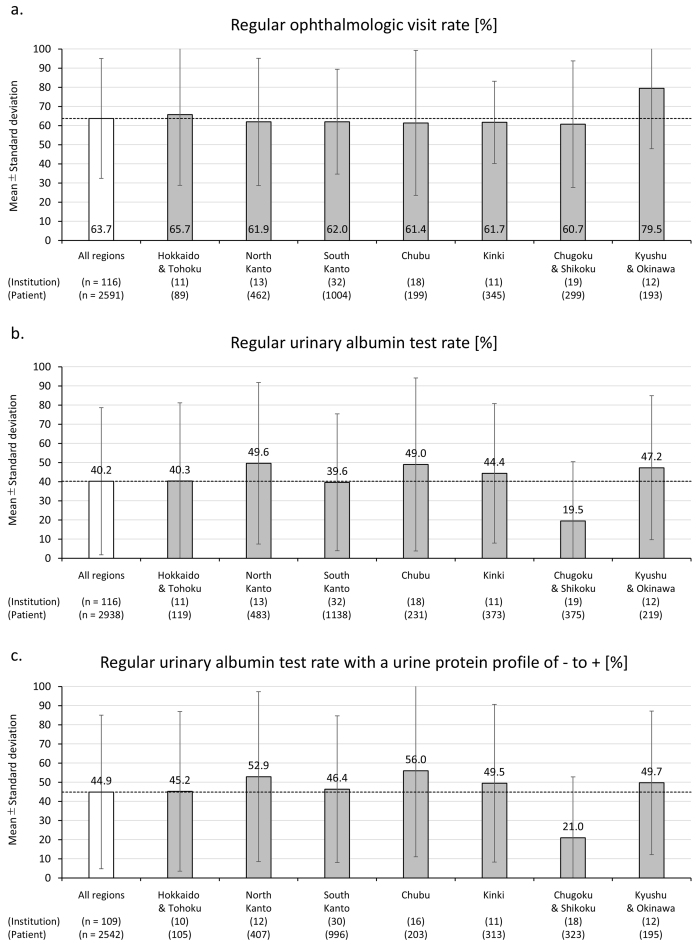
The bar graphs and error bars represent (a) the mean values (at the medical institution level) and standard deviations of the regular ophthalmology visit rate, (b) the urine albumin quantitative test implementation rate, and (c) likewise, among patients without macroproteinuria (*i.e.*, whose urine protein qualitative test is negative, trace (+/-), or 1+) by region. No statistically significant differences were detected in either the regular ophthalmology visit rate (*I*^2^ = 0.01%, p for heterogeneity = 0.778) (a), the urine albumin quantitative test implementation rate (*I*^2^ = 43.34%, p for heterogeneity = 0.124) (b), or *ditto* among patients without macroproteinuria (*I*^2^ = 48.17%, p for heterogeneity = 0.0758) (c).

The mean rates of regular ophthalmologic visits and urinary albumin quantification tests by region are shown in Figure 1. The mean ophthalmologic visit rate was 63.7% (SD: 31.3), with a median and interquartile range of 66.7% (42.7%-94.6%) (Figure 1a). The mean urinary albumin test rate was 40.2% (SD: 38.4), with a median and interquartile range of 31.7% (0.0%-76.4%) (Figure 1b). No statistically significant regional differences were found for either measure (ophthalmologic visit rate:* I*^2^ = 0.01%, p = 0.778; urinary albumin test rate: *I*^2^ = 43.34%, p = 0.124). Moreover, among patients without macroproteinuria (*i.e.*, those with a negative, trace, or 1+ urine protein qualitative test), the mean urinary albumin test rate was 44.9% (SD: 40.1), with a median and interquartile range of 44.4% (0.0%-87.5%). No statistically significant regional differences were observed in this subgroup either (*I*^2^ = 48.17%, p = 0.0758) (Figure 1c).

### Comparison between specialist and non-specialist institutions

The mean clinical parameters by specialist and non-specialist institutions are summarized in Table 3. The mean HbA1c level was significantly higher in specialist institutions (7.13% [SD: 0.41]) compared to non-specialist institutions (6.86% [SD: 0.46]; p = 0.001). No significant differences were found in systolic and diastolic blood pressure, LDL cholesterol, HDL cholesterol, or triglyceride levels between specialist and non-specialist institutions. The mean ophthalmologic visit rate was significantly higher in specialist institutions (78.5% [SD: 19.5]) than in non-specialist institutions (53.9% [SD: 33.8], p < 0.001). Similarly, the mean urinary albumin test rate among patients without macroproteinuria was significantly higher in specialist institutions (62.5% [SD: 35.5]) compared to non-specialist institutions (33.5% [SD: 39.0]; p < 0.001).

**Table 3.** Mean Clinical Parameters by Specialist and Non-Specialist Institutions.

Clinical parameters	Mean	SD	Median	IQR	p Value
HbA1c [NGSP] (%)					
Non-specialist institutions	6.86	0.46	7.0	6.6-7.1	Ref
Specialist institutions	7.13	0.41	7.1	6.8-7.3	0.001
Systolic blood pressure (mmHg)					
Non-specialist institutions	130.8	6.6	131	126-135	Ref
Specialist institutions	128.9	4.7	129	126-131	0.105
Diastolic blood pressure (mmHg)					
Non-specialist institutions	73.5	6.3	73	69-78	Ref
Specialist institutions	72.2	4.6	73	69-75	0.191
LDL cholesterol (mg/dL)					
Non-specialist institutions	105.2	14.0	106	95-112	Ref
Specialist institutions	106.2	9.8	105	100-111	0.664
HDL cholesterol (mg/dL)					
Non-specialist institutions	57.8	6.8	58	54-60	Ref
Specialist institutions	57.2	5.0	57	55-61	0.631
log Triglyceride (mg/dL)*					
Non-specialist institutions	2.11	0.11	136	121-171	Ref
Specialist institutions	2.12	0.07	152	131-167	0.510
Ophthalmology follow-up rate (%)					
Non-specialist institutions	53.9	33.8	50.0	32.0-88.1	Ref
Specialist institutions	78.5	19.5	80.5	63.4-100.0	<0.001
Urinary albumin quantification test rate (%)					
Non-specialist institutions	28.8	35.7	0.0	0.0-57.5	Ref
Specialist institutions	58.1	36.0	70.0	21.6-88.9	<0.001
Urinary albumin quantification test rate among patients without macroproteinuria (%)					
Non-specialist institutions	33.5	39.0	13.9	0.0-73.6	Ref
Specialist institutions	62.5	35.5	75.3	33.3-92.7	<0.001

p-value: unpaired Welch’s t-test. *The median and IQR for triglycerides are displayed as inverse log-transformed values (original scale). HbA1c: glycated hemoglobin; NGSP: National Glycohemoglobin Standardization Program; LDL: low-density lipoprotein; HDL: high-density lipoprotein; SD: standard deviation; IQR: interquartile range [25%-75%]; Ref: reference.

## Discussion

The regional comparison of clinical practices revealed no significant deviations from the national average in any of the analyzed regions, indicating a relatively uniform standard of diabetes care across Japan.

In contrast, significant differences were observed between specialist and non-specialist institutions in key clinical practices. The rate of regular ophthalmologic visits and the implementation rate of urinary albumin quantification tests were significantly higher in specialist institutions than in non-specialist institutions. Additionally, the mean HbA1c level was significantly higher in specialist institutions than in non-specialist institutions. This discrepancy in glycemic control suggests that specialist institutions tend to manage more complex diabetes cases that require specialized interventions and close monitoring, such as cases with a higher burden of comorbidities and/or difficulty achieving treatment goals, even with more intensive treatment.

Although there was a significant difference in HbA1c levels between specialist and non-specialist institutions, this was not the case with blood pressure or lipid profiles. As shown in Table 3, there were no statistically significant differences in blood pressure or lipid profiles. Taking this into account, it may well be possible that, with regard to blood pressure and lipid management, a greater portion of care is entrusted to non-specialist institutions compared to diabetes management.

As shown by Sugiyama et al. ^(6)^ using data from the National Database of Health Insurance Claims and Specific Health Checkups of Japan (NDB) for fiscal year 2015, retinopathy examinations were conducted among 46.5% of patients, whereas our current data (63.7%) exceeds this level. This higher implementation rate may be due to the fact that our analysis is based on data collected 7 years after the study by Sugiyama et al. ^(6)^, and that our data were obtained from a survey of medical institutions participating in J-DOME, which might introduce selection bias. On the other hand, the urinary quantitative protein or albumin examination rate reported by Sugiyama et al. ^(6)^ was 67.3%, while our rate (40.2%) is lower. However, it should be noted that the former figure includes measurements of urinary quantitative protein in addition to albumin. Considering that a portion of the former percentage is from urine protein measurement, our current results may be assessed within a similar range to the NDB data. In these regards, it is interesting and of note that ophthalmological assessment appears to be considerably acknowledged in the field of diabetes care.

### Limitations

As of the end of 2022, the total number of registered patients in the J-DOME database was just under 20,000 ^(7)^; by the end of 2024, this number is expected to reach 30,000 (unpublished data). However, this represents only a small fraction of the estimated 3.639 million individuals currently receiving treatment for type 2 diabetes in Japan, as reported in the 2023 Patient Survey by the Ministry of Health, Labour and Welfare ^(8)^. Given this limited sample size, expanding the database is necessary for more detailed comparisons and to enhance the generalizability of the findings. In particular, we should be very careful in interpreting data from areas with fewer participating institutions, such as Hokkaido/Tohoku and Kinki, due to potential biases caused by the limited sample size.

Although the observed differences between specialist and non-specialist institutions are intriguing, our registry has only limited entries regarding therapy, such as types of drugs administered, whether or not dietary therapy by a registered dietitian was introduced, and likewise, exercise therapy. For these items, we have found only a little difference between specialist and non-specialist institutions in the registry as a whole at that time ^(9)^ and we have been unable to elucidate the reasons for the above-described differences.

### Future perspective

As mentioned above, we do not have sufficient data on psychological care. However, precisely because of this background, collaboration with multidisciplinary healthcare professionals, which is definitely important for diabetes care, should be assessed in forthcoming protocols.

Future studies should consider stratifying analyses by secondary medical care areas, taking into account regional characteristics such as metropolitan areas, regional cities, and rural or depopulated areas. This approach could provide deeper insights into potential disparities in diabetes care across various healthcare settings.

## Article Information

### Acknowledgments

The authors express the deepest gratitude to all medical institutions participating in the J-DOME database and to all patients who contributed to this study through their registrations. We also express our profound gratitude to the Practitioners Subcommittee in the Japanese Society of Hypertension for their contributions. In addition, we thank Drs. Norihito Kamimura (Kamimura Clinic), Yoshihisa Takada (Takada Clinic), Masahiro Fukuda (Fukuda Clinic), Hidekatsu Sugimoto (Sugimoto Clinic), Tetsuro Tsujimoto (Toranomon Hospital Kajigaya), Iseki Takamoto (Tokyo Medical University), Kei Asayama (Teikyo University School of Medicine), and Tomohiro Katsuya (Katsuya Clinic) for their collaborative participation in this work.

### Author Contributions

Mitsuhiko Noda and Atsushi Goto contributed to the analysis of the data and to the writing of the manuscript. Kohjiro Ueki, Koichi Node, Hiromi Rakugi, and Narumi Eguchi interpreted the results and the supervision of the manuscript. Mitsuhiko Noda and Narumi Eguchi contributed to the design and implementation of the study. All authors discussed the results and contributed to the final manuscript.

### Conflicts of Interest

None

### Ethical Approval

This study was approved by the Japan Medical Association Ethical Review Board (28-3-4).

### Disclaimer

Kohjiro Ueki and Narumi Eguchi are the Editors of JMA Journal and on the journal’s Editorial Staff. They were not involved in the editorial evaluation or decision to accept this article for publication at all.
